# Infection with SARS-CoV-2 variant Gamma (P.1) in Chile increased ICU admission risk three to five-fold

**DOI:** 10.1371/journal.pone.0283085

**Published:** 2023-03-24

**Authors:** Denis Sauré, Ignasi Neira, Marcel Goic, Miguel O’Ryan, Juan P. Torres, Alejandro Bruhn, Marcela Ferres, Jenniffer Angulo, Magdalena Vera, Leonardo J. Basso

**Affiliations:** 1 Industrial Engineering Department, Faculty of Physical and Mathematical Sciences, Universidad de Chile, Santiago, Chile; 2 Instituto Sistemas Complejos de Ingeniería (ISCI), Santiago, Chile; 3 Instituto de Ciencias Biomédicas, Faculty of Medicine, Universidad de Chile, Santiago, Chile; 4 Department of Pediatrics and Pediatric Surgery, Faculty of Medicine, Universidad de Chile, Santiago, Chile; 5 Department of Intensive Care Medicine, School of Medicine, Pontificia Universidad Católica de Chile, Santiago, Chile; 6 Department of Infectious Diseases and Pediatric Immunology, School of Medicine, Pontificia Universidad Católica de Chile, Santiago, Chile; 7 Laboratorio Infectología y Virología Molecular, School of Medicine, Pontificia Universidad Católica de Chile, Santiago, Chile; 8 Civil Engineering Department, Faculty of Physical and Mathematical Sciences, Universidad de Chile, Santiago, Chile; Politecnico di Torino, ITALY

## Abstract

The 2021 wave of SARS-CoV-2 infection in Chile was characterized by an explosive increase in ICU admissions, which disproportionately affected individuals younger than 60 years. This second wave was also accompanied by an explosive increase in Gamma (P.1) variant detections and the massive vaccine rollout. We unveil the role the Gamma variant played in stressing the use of critical care, by developing and calibrating a queueing model that uses data on new onset cases and actual ICU occupancy, symptom’s onset to ICU admission interval, ICU length-of-stay, genomic surveillance, and vaccine effectiveness. Our model shows that infection with the Gamma (P.1) variant led to a 3.5–4.7-fold increase in ICU admission for people younger than 60 years. This situation occurred on top of the already reported higher infection rate of the Gamma variant. Importantly, our results also strongly suggest that the vaccines used in Chile (inactivated mostly, but also an mRNA), were able to curb Gamma variant ICU admission over infections.

## Introduction

### Background and motivation

COVID-19 associated intensive care unit (ICU) occupancy has been strictly monitored in Chile throughout the pandemic. Occupancy rates reached 90% by mid 2020, during Chile’s first wave, and remained between 75%-90% between July and December 2020 [1]. From January 2021 onwards, ICU occupancy surpassed 90%, and progressively increased until reaching 99% in June, despite a nearly four-fold increase in the number of ICU beds during this period. This explosive increase in ICU admissions was characterized by a disproportionately higher rate of ICU admissions over new infections, compared to that observed during 2020, particularly for individuals younger than 60 years of age. In addition, data from monitoring of variants of concern (VOCs) showed that during 2021 the Gamma (P.1) variant had increased from no detections in January to a proportion of over 65% by mid-June, as it did in the rest of Latin America; the Alpha (B.117) variant in contrast, reached a proportion of 10% by March, almost disappearing (3%) by May.

**Fig 1:**
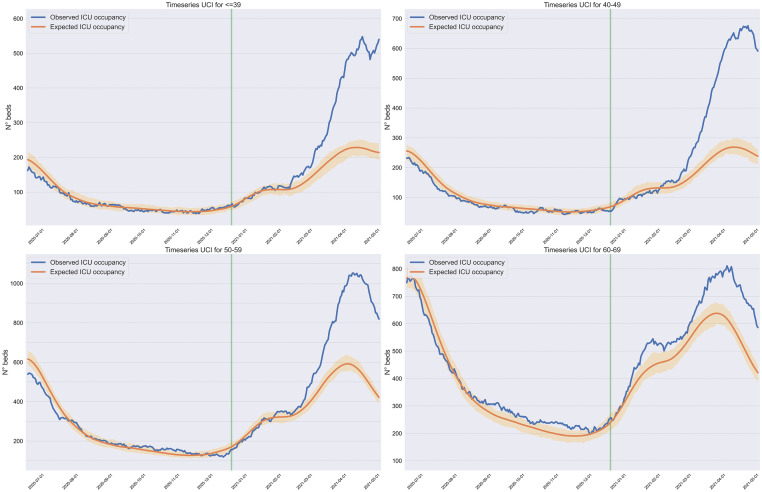
Actual and predicted (expected) ICU occupancy by age, 2020-model. The blue and orange curves depict the actual and predicted (using the 2020-model) ICU occupancy, and the shaded area represents the 95% confidence intervals for the predicted ICU occupancy. The green vertical line denotes the beginning of 2021.

In May 2020, a prediction model for COVID-19 associated ICU occupancy was developed, based on an ensemble of models, in order to predict occupancy rates up to two weeks in advance, with the purpose of supporting the government strategy of dynamically adapting the country’s public-private joint ICU capacity [2]. One of the models used was a queuing model of ICU occupancy based on data of new symptomatic cases, ICU occupancy, symptom onset to ICU admission interval and ICU length-of-stay. The model, which henceforth we refer to as the 2020-model, was capable of reconstructing with minimum error the nation-wide ICU occupancy time-series during 2020. However, the queuing model applied to 2021 data evidenced a fundamental change in the predictive capacity of ICU occupancy with significantly lower ICU predicted admissions than those observed. This discrepancy between predicted and observed ICU occupancy, which can be observed in Fig 1 and is the central object of interest in our research, together with the incoming information on the circulation of VOCs [3] and data on vaccine effectiveness in Chile [4], suggested that the Gamma variant was significantly increasing the risk of ICU admissions conditional on infection; increased transmissibility was an unlikely explanation because the model takes new symptomatic cases as input.

The hypothesis in this study is that it is possible to estimate the increase in ICU admission rate associated with the Gamma variant, and that the resulting estimates are significantly higher for younger age brackets. We test this hypothesis by extending the 2020-model to account for variant circulation, and vaccine effectiveness when necessary, in such a way that the only parameter that remains to be estimated from available data and the model are age-dependent relative risks of ICU admission associated with the Gamma variant.

### Overall strategy

**Fig 2:**
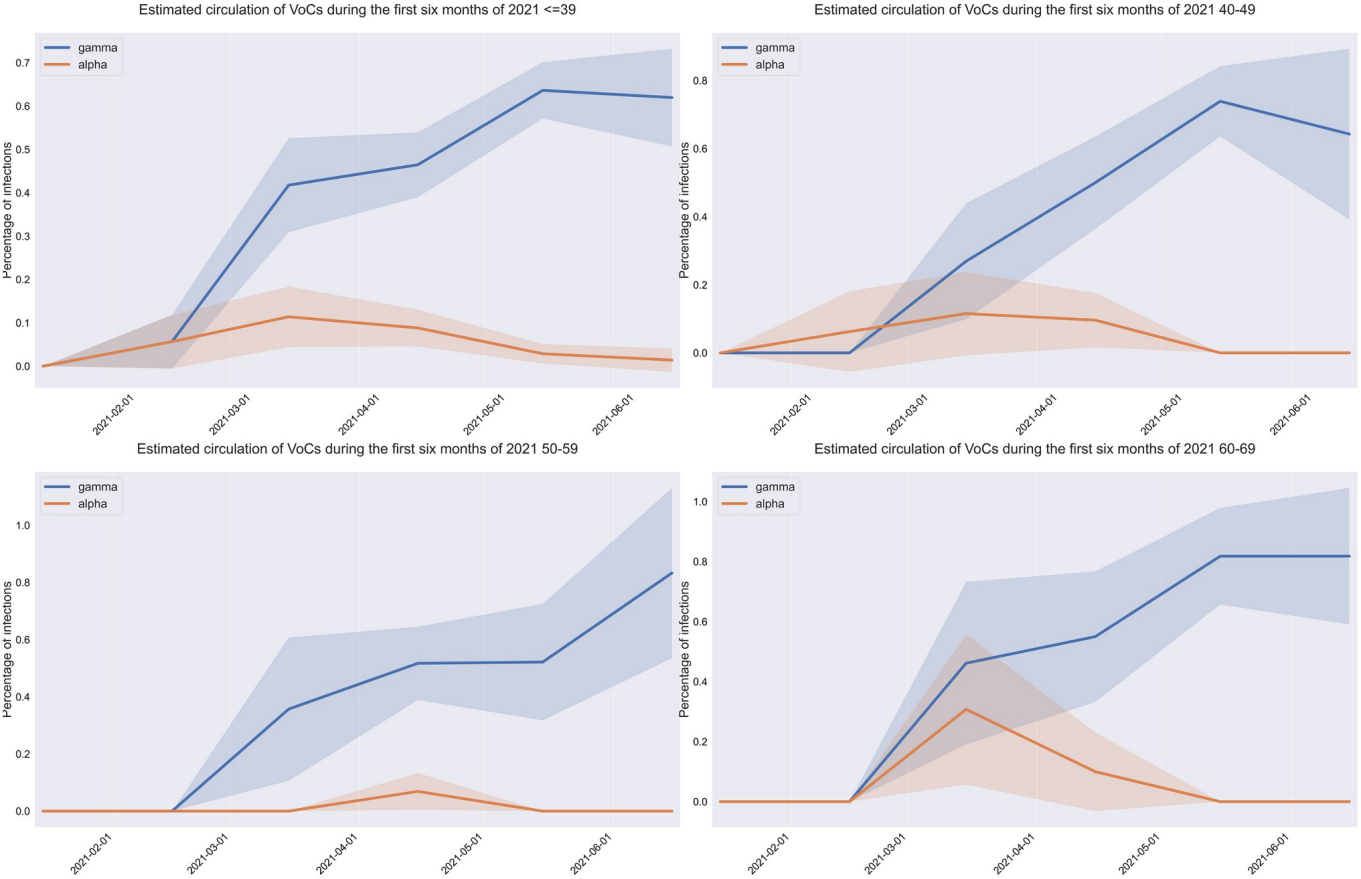
Estimated circulation of VOCs during the first five months of 2021. The blue and red curves depict the estimated percentage of infections associated with the Gamma and Beta variants, respectively, from January through May, 2021. The shaded area represents 95% confidence intervals for these estimates.

The estimation strategy is the following: we calibrated the 2020-model with 2020 data when circulation of VOCs was still absent. Then, we expanded the model to include the effect of vaccine rollout which was characterized by a rapid onset in the elder population beginning in February 2021, followed by a decreasing-age strategy [5]. The need to disentangle the effects of variants and vaccination rollout forced us to exclude subjects 70 years and older from the analysis. We then applied the resulting version of the 2020-model (that did not differentiate by the type of SARS-CoV-2 variant) to 2021 data, which resulted in a large discrepancy between predicted and actual ICU occupancies. Subsequently, we refined the model so as to solve the discrepancy using genomic surveillance information (see Fig 2) and by introducing different parameters possibly related to the variant; the resulting model, which we refer to as the 2021-model, resulted in the estimation of increased risk of ICU admission for symptomatic infections due to the Gamma variant. In addition to the five parameters used by the 2020-model, the 2021-model requires and incorporates three additional sets of parameters: the circulation of VOCs, the effectiveness of the vaccines from Sinovac and Pfizer, and the probability of ICU admission for symptomatic infection for the Gamma variant, which is our estimation and main result.

**Fig 3:**
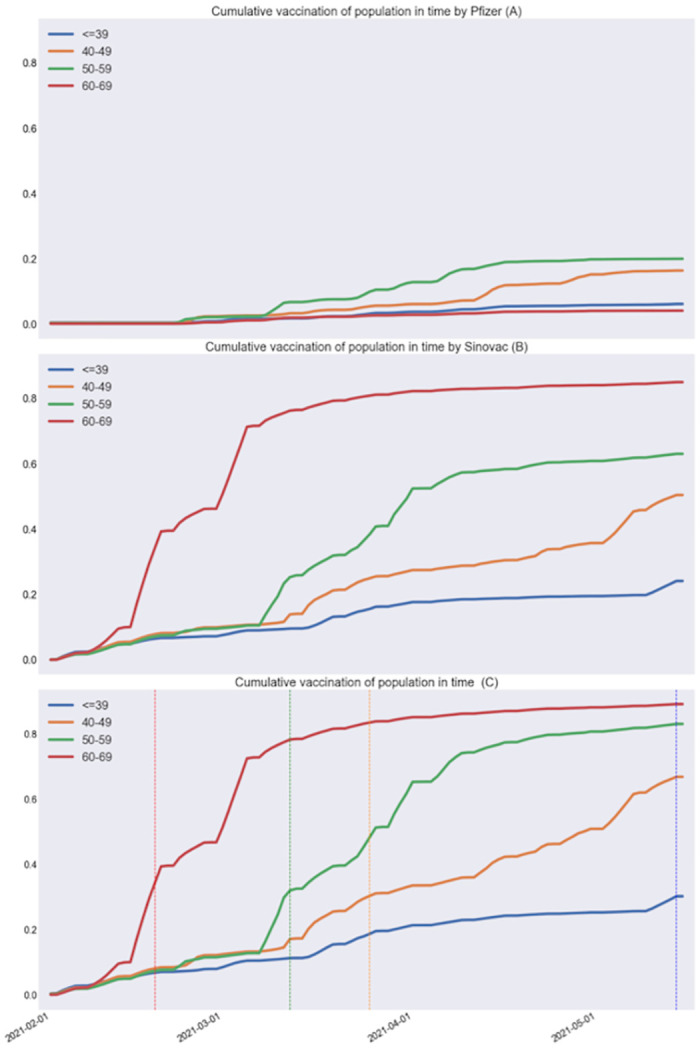
Massive vaccination rollout. For each age-bracket, curves in **(A)** and **(B)** depict the cumulative vaccination (first doses) for the mRNA vaccine from Pfizer and the inactivated virus vaccine from Sinovac, respectively, as a percentage of the eligible population. **(C)** depicts the total cumulative vaccination, where for each age bracket, a colored vertical line indicates the date on which accumulative vaccination reached 30% of the eligible population.

The 2020-model and 2021-model are stochastic models corresponding to two uncapacitated queues in tandem. For each age bracket, the models take the time-series of new symptomatic cases and then use a probability of ICU admission and a probabilistic distribution of time to admission from symptom onset (first queue). They then use a probabilistic distribution—per age bracket—of length of stay in ICU (second queue). These probabilities needed to be estimated or calibrated. Vaccination status of new cases were estimated based on the vaccine rollout (Fig 3) while vaccine effectiveness was estimated based on national reports [4], both using Bayes formula: See S1 Appendix for the details.

The models used as an input a constructed nation-wide time-series of symptomatic infections by age from publicly available data [6], thus eliminating the need to model transmission dynamics to forecast new cases and capturing all infections, including those caused by VOCs. The interval between infection onset to ICU admission and ICU length-of-stay were modeled and estimated using data from one of Chile’s largest ICU wards, at the Hospital Clínico of the Pontificia Universidad Católica. This data set includes ages, dates of infection onset, ICU admission and discharge, for 247 patients admitted between March 2020 and May 2021: see Table 1. For the 89 patients in the data set admitted after February 2021, the dataset included in addition virus sequencing results identifying Gamma and Alpha variants.

**Table 1:** Summary of data on onset-to-ICU interval and ICU length-of-stay times.

Age-bracket	N° obs 2020	N° obs 2021		Mean onset-to-ICU (95% CI)	Mean ICU length-of-stay (95% CI)
		non-censored	censored		
< = 39	20	12	5	7.75 (5.55, 9.94)	25.20 (14.64, 35.75)
40–49	19	15	9	10.31 (7.97, 12.65)	25.94 (14.99, 36.90)
50–59	54	18	13	9.79 (7.84, 11.74)	22.20 (16.92, 27.48)
60–69	65	10	7	12.64 (9.97, 15.31)	26.60 (20.95, 32.24)

In the 2020-model, the probabilities of ICU admission conditional on symptomatic infection were estimated using Little’s Law [7], that relates mean occupancy, arrival rate and length-of-stay in queuing systems. Regarding vaccination, by June 17 2021, 77.8% of the eligible population had been vaccinated with a first dose, out of which 76% were vaccinated with the inactivated virus vaccine from Sinovac and 19% with the mRNA vaccine from Pfizer [5]. The model allows specific vaccination status imputations based on the detailed data available for the rollout and effectiveness [4] of the two vaccines.

For the 2021-model, the VOCs circulation curves depicted in Fig 2 were estimated using data from genomic surveillance performed in outpatients by the Laboratorio de Virología Molecular de la Pontificia Universidad Católica. The data set contains age, date and outcome of the sequencing test for roughly 900 patients positive for SARS-COV-2 between January and June 2021 (see S1 Table), and shows that only two VOCs were significantly present, namely the Alpha and Gamma variants. The shape of VOCs circulation curves depicted in Fig 2 are aligned with the age-aggregated curve provided by the national genomic surveillance data [3]. Averaging over all age brackets, the Gamma variant increased from 3.2% (CI 2.6%–3.8%) in February 2021 to 66.1% (CI 63.6%–68.5%) in May 2021. See S2 Appendix for more details.

For the Alpha variant, we used estimates from the literature [8, 9], which concluded a relative risk associated with ICU admission of 2.03 (CI 1.69, 2.45) when compared to the original virus, across all ages. For the Gamma variant, we estimated the relative risk for ICU admission independently for each age range, by minimizing the mean square error between the 2021-model and the observed occupancy curves during 2021.

## Methods

### The two tandem-queues ICU occupation model

#### The 2020-model

The 2020-model for ICU occupation receives as an input the nation-wide time-series $\left\{n_{t}^{a}:t\leq T\right\}$ of symptomatic infections, were $n_{t}^{a}$ denotes the number of people within age bracket *a* ∈ *A* that begin symptom onset on day *t*, *T* denotes the length of the horizon (April 1th 2020 to May 15th 2021, in days), and *A* denotes the age brackets considered. Someone in age bracket *a* beginning symptom onset in day *t* requires ICU admission with probability $p_{ICU}^{a}$ independent of other factors; if the patient requires ICU admission, this occurs $X_{adm}^{a}$ days after symptom onset; the patient stays at the ICU for $X_{ICU}^{a}$ days before being discharged. We assumed that, for each age bracket *a*, $X_{\;adm}^{a}$ and $X_{ICU}^{a}$ are random variables, independent across days and patients, and such that

$$X_{adm}^{a}∼\text{NegativeBinomial}\left(\mu_{adm}^{a},r_{adm}^{a}\right),\;\text{and}\;X_{ICU}^{a}∼\text{NegativeBinomial}\left(\mu_{ICU}^{a},r_{ICU}^{a}\right).$$

For a given set of parameters $\left(p_{ICU}^{a},\mu_{adm}^{a},r_{adm}^{a},\mu_{ICU}^{a},r_{ICU}^{a}:a\in A\right)$, under the assumption of no infections prior to *t* = 0, the expected number of ICU beds occupied on day *t* by patients in age bracket *a* according to our model, ${\hat{b}}_{t}^{a}$, is given by

$${\hat{b}}_{t}^{a}=\;\underset{s<t}{\sum }\;n_{s}^{a}\cdot p_{ICU}^{a}\cdot \underset{h<t-s}{\sum }\text{Pr}\left(X_{adm}^{a}=h\right)\cdot \text{Pr}(X_{ICU}^{a}>t-s-h)\;.$$

The standard deviation of ${\hat{b}}_{t}^{a}$ is easily computed using the independence assumption, which allows estimations of confidence intervals for the series of number of ICU beds occupied.

#### The 2021-model

The overall evolution of the symptomatic individual remained as in the original model, with the exception that parameters governing onset-to-ICU time interval, ICU length-of-stay distributions, and the probability of ICU admission conditional on symptomatic infection were allowed to potentially depend on the infecting variant. Regarding vaccination, it is assumed that vaccine effectiveness for VOCs is not different from that reported against the background strain, and that protection remained constant after two weeks of second doses, and we conducted vaccine status imputations using vaccination rollout data [5], adjusting ICU admission probabilities accordingly; this assumption is further discussed below.

To accommodate the role of the variants in ICU occupation, we modified the dynamics as follows. We consider that a patient *n*, from a fixed age bracket *a* and day *t* $\left(n\leq n_{t}^{a}\right)$, was infected with the VOC *m* ∈ *M* with probability $q_{t}^{a,m}$, where the set *M* includes the Alpha and Gamma variants, and the background (and possibly others) strain. Thus, allowing $v_{n,t}^{a}$ to denote the (non-) variant associated with patient *n*, we have that

$$v_{nt}^{a}∼\text{Categorical}\left(q_{t}^{a,0},q_{t}^{a,Alpha},q_{t}^{a,Gamma}\right),\;\;n\leq \;n_{t}^{a},\;t\leq T$$

In addition, the probability that someone with a symptomatic infection requires ICU admission is given by $p_{ICU}^{a,m}$, i.e. depends on the variant associated with the patient. Thus, in addition to the parameters in the original ICU occupation model, the adjusted model is defined by a circulation curve $\left(q_{t}^{a,m},\;t\leq T,\;m\in M,\;a\in A\right)$, and the variant dependent probabilities $\left(p_{ICU}^{a,m},\;a\in A,\;m\in M\right)$.

The time and age-dependent variant circulation curves used in the 2021-model are those depicted in Fig 2, which were obtained from genomic surveillance.

### Calibration of onset to ICU and ICU length-of-stay times

In our models, we assumed that the number of days from symptoms onset to ICU admission and the duration in ICU are both distributed according to Negative Binomial distributions. Given a data set of durations, we estimated the mean μ and dispersion *r* of the distribution by maximizing the log-likelihood of the observed data. We assume that durations are independent by age bracket. Under this assumption, the log-likelihood is additively separable by age bracket, and the Fisher information matrix associated with the durations has an analytical form. This allows us to construct confidence intervals for the parameters using a Normal (asymptotic) approximation.

Our first analysis focused on potential differences on onset of symptoms-to-ICU and ICU length-of-stay time distributions for VOCs, as compared to the pre-VOCs period. Based on the literature [10], we hypothesized that onset-to-ICU and ICU length-of-stay time distributions remained unchanged for the relevant VOCs and that they were the same before and after January 2021. We tested these hypotheses using a likelihood-ratio test, because the length-of-stay information was censored for the latest entries in the data set (patients remaining in the ICU) and thus biased towards patients infected with the Gamma variant; this procedure has been previously used for censored data in the context of COVID-19 hospitalizations [11].

See S3 Appendix for more details on the estimation and hypothesis testing procedures.

Having calibrated these distributions, the only remaining possible explanation for the mismatch depicted in Fig 1, between expected (according to the queuing model) and observed ICU occupancies prior to vaccine rollout, was a difference in probabilities of ICU admission conditional on symptomatic infection by one of the VOCs.

### Construction of time-series of symptomatic infections by age

We construct a nation-wide times-series of symptomatic infections by age bracket as follows. First, we use the nation-wide time-series of symptomatic infections (Product 26, at [6]) and asymptomatic infections (Product 27, at [6]) to estimate a time-series of symptomatic to asymptomatic infections ratios. Then, assuming the ratio between symptomatic and asymptomatic infections is homogeneous across age brackets (we further discuss this assumption later on, in our sensitivity analysis), we use such a time-series and the nation-wide time-series of infections by age bracket (Product 16, at [6]), to produce a time-series of asymptomatic infections by age bracket. Finally, because entries in the constructed time-series are registered by the time infections are confirmed via RT-PCR tests, for each age bracket, we shift the time-series a number of days equal to the mean gap between symptom onset and confirmation date, computed using data from the Laboratorio Infectologia y Virologia Molecular of the School of Medicine of the Pontificia Universidad Católica de Chile.

### Estimation of ICU admission conditional on original strain infection

For age bracket *a* ∈ *A*, let L^*a*^(t) denote the number of patients that at time *t* occupy an ICU bed, and let N^*a*^(t) denote the cumulative number of symptomatic infections by time *t*. Applied to our ICU occupancy system, Little’s Law [7] states that if the time-average limits L^*a*^ and *λ*^***α***^ of L^*a*^(t) and N^*a*^(t) exist, then

$$L^{a}=\;\lambda^{a}\cdot p_{ICU}^{a}\cdot \mu_{ICU}^{a}.$$

From this relation, we estimated the probability of ICU admission conditional on infection as

$${\hat{p}}_{ICU}^{a}=\frac{{\bar{L}}_{\left(t_{i}^{a},t_{f}^{a}\right)}^{a}}{\;{\bar{\lambda }}_{\left(t_{i}^{a},t_{f}^{a}\;\right)}^{a}\cdot \mu_{ICU}^{a}},$$

where ${\overset{-}{L}}_{\left(t_{i}^{a},t_{f}^{a}\right)}^{a}$ and ${\overset{-}{\lambda }}_{\left(t_{i}^{a},t_{f}^{a}\;\right)}^{a}$ denote the average ICU occupancy and new cases per day between days $t_{i}^{a}$ and $t_{f}^{a}$, respectively. This probability estimate assumes the existence of the limiting time-averages and relies on an asymptotic approximation for both the limiting time-average ICU occupancy and admission rate. We considered time-windows such that both daily new cases and ICU occupancy were rather stable. For each time window, we considered December 31th, 2020 as the final day $t_{f}^{a}$, and varied the starting date $t_{i}^{a}$. S2 Table shows the mean square errors between the expected and observed ICU occupancy during 2020 as a function of the starting date. Considering these results, we used May 2020 as the starting date across all age brackets.

### Estimation of ICU admission conditional on Gamma infection

All model parameters for the 2020-model are estimated from available data. For the 2021-model this is also the case, with the exception of the probabilities of ICU admission after symptomatic infection with the Gamma variant. Note that these probabilities could not be derived from Little’s Law because of the transient nature of the VOC irruption. Instead, we estimated such probabilities by minimizing the mean square error between the expected and observed ICU admission. For each age bracket we solved

$${\text{min}}_{p_{\text{ICU}}^{a,\text{Gamma}}}\sum_{t=t_{i}^{a}}^{t=t_{f}^{a}}{\left({\hat{b}}_{t}^{a}-b_{t}^{a}\right)}^{2},$$

Where $t_{i}^{a}$ and $t_{f}^{a}$ denote January 1 and May 15, 2021, respectively, for all age brackets.

## Results

In our analysis, we found no statistical difference on onset-to-ICU interval and ICU length-of-stay distribution parameters between the original virus and the VOCs (see Table 2A); this was also the case for ICU length-of-stay between time periods before and after January 2021 (see Table 2B). Our analysis compared the likelihood of a model that restricted the distribution parameters to be the same across VOC and/or time periods against that of an unrestricted (full) model. Considering this, we proceeded to estimate separate parameters for the Onset to ICU distribution for 2020 and 2021, and a single set of parameters for the ICU length-of-stay distribution: see Table 3. These estimates are in line with other reports [10].

**Table 2:** Likelihood Ratio Test: Equal distribution of Onset-to-ICU and ICU length-of-stay distributions for (A) VOCs and background strain and (B) before and after January 2021. Entries depict either parameter estimates (and CI) and log likelihood (LL) for each model.

**(A) VOCs and background strain**.			Full model	Restricted model
onset-to-ICU	VOC	mean (μ)	9.395 (8.293, 10.497)	9.395 (8.545, 10.246)
		dispersion (r)	12.00 (11.996, 12.004)	12.00 (11.997, 12.003)
	O. strain	mean (μ)	8.905 (7.402, 10.409)	9.395 (8.545, 10.246)
		dispersion (r)	6.457 (6.454, 6.461)	12.00 (11.997, 12.003)
		LL	246.018	247.487
ICU length-of-stay	VOC	mean (μ)	34.683 (28.173, 41.195)	30.489 (23.075, 37.904)
		dispersion (r)	2.102 (2.100, 2.104)	1.918 (1.916, 1.920)
	O. strain	mean (μ)	25.684 (19.103, 32.267)	30.489 (23.075, 37.904)
		dispersion (r)	1.734 (1.733, 1.736)	1.918 (1.916, 1.920)
		LL	234.75	236.27
**(B) Before and after January 2021**			Full model	Restricted model
onset-to-ICU	2021	mean (μ)	9.395 (8.545, 10.246)	9.997 (9.098, 10.898)
		dispersion (r)	12.00 (11.997, 12.003)	4.326 (4.325, 4.328)
	2020	mean (μ)	10.349 (9.301, 11.397)	9.997 (9.098, 10.898)
		dispersion (r)	3.102 (3.101, 3.103)	4.326 (4.325, 4.328)
		LL	749.669	759.916
ICU length-of-stay	2021	mean (μ)	30.489 (23.075, 37.904)	26.598 (23.349, 29.849)
		dispersion	1.918 (1.916, 1.920)	1.735 (1.734, 1.736)
	2020	mean (μ)	24.655 (21.473, 27.839)	26.598 (23.349, 29.849)
		dispersion (r)	1.551 (1.55, 1.552)	1.735 (1.734, 1.736)
		LL	898.371	901.066

**Table 3:** Estimates for parameters of the onset-to-ICU interval and ICU length-of-stay distributions by age bracket. Estimates for the mean (μ) and dispersion (r) parameters of the negative binomial distribution for onset-to-ICU interval and ICU length-of-stay time periods, and for the probability of ICU admission conditional to infection. Brackets indicated 95% confidence intervals.

Age-bracket	Onset to ICU 2020		Onset to ICU 2021		ICU length-of-stay		Prob. ICU adm. given infection
	mean (μ)	dispersion (r)	mean (μ)	dispersion (r)	mean (μ)	dispersion (r)	
< = 39	7.74	5.11	8.91	9.20	25.19	2.01	0.33%
	(5.81, 9.69)	(5.10, 5.11)	(7.30, 10.52)	(9.19, 9.20)	(17.09, 33.28)	(2.00, 2.01)	(0.25%, 0.49%)
40–49	10.31	8.88	9.38	21.79	25.94	1.94	1.18%
	(8.18, 12.43)	(8.88, 8.89)	(7.92, 10.85)	(21.78, 21.80)	(17.27, 34.60)	(1.94, 1.95)	(0.85%, 1.71%)
50–59	9.80	2.66	9.33	6.27	22.19	1.46	3.59%
	(7.99, 11.60)	(2.66, 2.66)	(7.60, 11.05)	(6.26, 6.27)	(17.14, 27.24)	(1.46, 1.46)	(2.90%, 4.61%)
60–69	12.65	2.22	11.42	6.56	26.60	1.44	7.18%
	(10.41, 14.88)	(2.22, 2.22)	(8.68, 14.16)	(6.56, 6.57)	(21.07, 32.12)	(1.44, 1.44)	(5.81%, 8.86%)

Fig 1 shows that the 2020-model, estimated separately for each age bracket (orange lines), reconstruct well the observed time-series of ICU occupancy (blue lines) from July 2020 to February 2021, for individuals 69 years and younger (see S3 Table for statistical information on goodness of fit); results prior to June 2020 were excluded because data on infections prior to April 2020 was scarce and thus less reliable.

From Fig 1, we observed that starting in February-March 2021, the ICU occupancy projected by the 2020-model diverges heavily from the observed occupancy curve for all age brackets with the exception of the 60–69 years age bracket. Considering this, we hypothesized that the irruption of VOCs was primarily responsible for this divergence, and proceeded to calibrate the 2021-model.

**Fig 4:**
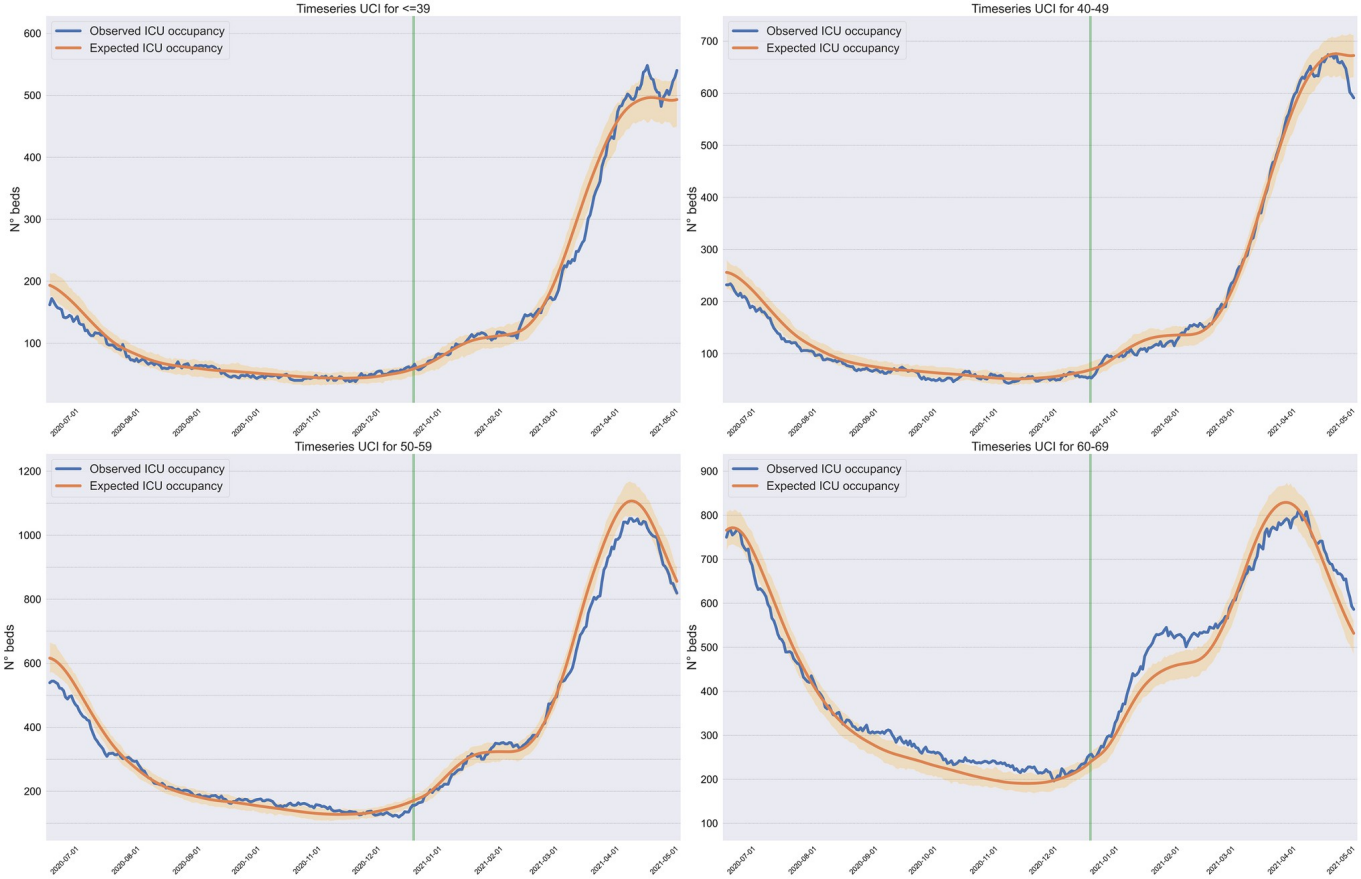
Actual and predicted (expected) ICU occupancy by age during 2020 and 2021, 2021-model. The blue and orange curves depict the observed and predicted (using the 2021-model) ICU occupancy, and the shaded area represents 95% confidence intervals for the expected ICU occupancy.

Fig 4 depicts the results of our estimation procedure. The adjusted queuing model now had the capacity to reconstruct the observed ICU occupancy curve during the complete period, including February 2020 onwards (see S3 Table for statistics on goodness of fit).

Table 4 shows the estimates for the relative risk for ICU admission associated with the Gamma variant, i.e. how many times more likely is that a person infected with the Gamma variant, and who develop symptoms, ends up admitted to an ICU relative to the original virus. Confidence intervals for the relative risk are constructed considering the uncertainty surrounding the various estimated parameters used in our model.

**Table 4:** Relative risk estimates (and confidence intervals) by age for ICU admission conditional on infection with the Gamma variant.

	< = 39	40–49	50–59	60–69
Prob (ICU|infected with Gamma)	1.15% (0.95%, 1.36%)	5.30% (4.00%, 7.57%)	11.17% (7.07%, 14.58%)	8.90% (5.09%, 12.71%)
Gamma Relative Risk	3.46 (2.86, 4.09)	4.67 (3.39, 6.42)	3.67 (1.97, 4.06)	1.24 (0.71, 1.77)

### Sensitivity analysis: Disentangling the effect of vaccination

Considering that the 2021 expected ICU occupancy curve depended on our assumption on similar vaccine effectiveness for all VOCs, we conducted the following sensitivity analysis: for each bracket we repeated the estimation procedure above, but setting $t_{f}^{a}$ (the final date in the time window used for estimating the probability of ICU admission conditional on original virus infection for age bracket *a*) to two weeks after the date by which the first dose vaccine rollout reached 30% of the eligible population; the premise here is that, because effectiveness of the inactivated virus vaccine is low prior to the second dose, and that time from onset to ICU is in average over a week, the effect of vaccine rollout on ICU occupancy by the aforementioned milestone should be small. Unfortunately, for the 60–69 age bracket this alternative estimation was not feasible, because circulation of variants was not large enough to enable identification (that is, the mean square error was insensitive to changes in the risk factor). The alternative estimated parameters are shown in Table 5; note that they differ slightly from those depicted in Table 4, and that the mean square error is less sensitive to changes in the parameter for older age brackets, signaling that for such brackets is it harder to disentangle the effects of VOC circulation and vaccine rollout.

**Table 5:** Alternative relative risk estimates (and confidence intervals) for ICU admission conditional to infection by age for the Gamma variant—Computed using age-dependent time-windows, minimizing mean square error.

	Age range		
	< = 39	40–49	50–59
Pr(UCI|cont. w Gamma.)	1.15% (0.95%, 1.36%)	5.52% (3.53%, 8.28%)	12.59% (6.30%, 18.89%)
Gamma Relative Risk	3.46 (2.86, 4.09)	4.87 (2.99, 7.02)	4.14 (1.75, 5.26)

We observe that the alternative estimates—which minimize the mean square error between the observed and predicted occupancy curves—are larger than but close to those in Table 4, reaching risk factors of 4.87 for people in the 40 to 49 age bracket and 4.14 for people in the 50 to 59-year bracket. This speaks, first, of the robustness of our results and, second, that the assumption on similar vaccine effectiveness is not unreasonable.

### Sensitivity analysis: The effect of heterogeneous symptomatic-to-asymptomatic infection ratios

Both the 2020-model and the 2021-model requires as input the series of symptomatic infections by age bracket. Due to the lack of symptomatic infection data disaggregated by age, we constructed the required series from available total infection data under the assumption of a homogeneous symptomatic-to-asymptomatic infection ratios across age brackets. Considering evidence pointing to heterogeneity is those ratios [12], we conduct the following sensitivity analysis: we borrow estimates of symptomatic-to-asymptomatic ratios by age bracket from the literature [13] and construct a new series of symptomatic infections by age using these ratios as fixed and using the value of the youngest age bracket as a reference. Then, we repeat the estimation of the 2020-model and 2021-model, but using these new inputs. The alternative estimated parameters are shown in Table 6.

**Table 6:** Alternative relative risk and probabilities of ICU admission conditional on symptomatic infection (and confidence intervals) by age using age-dependent symptomatic-to-asymptomatic ratios.

	< = 39	40–49	50–59	60–69
Relative Simptomatic-to-Asymptomatic ratio	1.0 (reference)	1.03	1.04	0.99
Prob (ICU|infected with o. strain)	0.34% (0.26%, 0.49%)	1.16% (0.85%, 1.70%)	3.46% (2.81%, 4.58%)	7.31% (5.85%, 8.90%)
Prob (ICU|infected with Gamma)	1.18% (0.99, 1.38)	3.90% (3.20, 6.51)	8.60% (7.34, 12.81)	9.15% (4.97, 12.79)
Gamma Relative Risk	3.46 (2.93, 4.11)	3.36 (2.76, 5.61)	2.48 (2.12, 3.70)	1.25 (0.68, 1.75)

Compared to the results of the main model presented in Table 4, there is a slight increase in the relative risk of the Gamma variant associated with individuals younger than 39 years (who concentrate the largest amount of symptomatic infections during 2021) and a more pronounced decrease in the values associated with individuals ages between 40 and 59 years. Overall, these results indicate that even if we consider heterogeneous symptomatic-to-asymptomatic infection ratios, the Gamma variant is associated to larger relative risk, especially for the younger age brackets.

## Discussion

Starting in February-March 2021, the ICU occupancy projected by the 2020-model diverges heavily from the observed occupancy curve for all age brackets with the exception of the 60–69 years age bracket. For the other three age brackets, divergence increases with time, most notably in the younger age brackets. Vaccination reached 20% for the 60-69-year age bracket in mid-February 2021 while for the population below 39 years of age this coverage was reached only by April 2021. This change in underlying dynamics of the pandemic, including age heterogeneity, can also be observed from the nation-wide raw data on new cases time-series (See S1 Fig).

Considering the above, we hypothesized that the irruption of VOCs, particularly Gamma, was primarily responsible for the abrupt change in ICU occupancy dynamics motivating an adaptation to the tandem-queues model to accommodate the 2021 irruption of VOCs. As a result of calibrating the 2021-model, the estimated relative risk associated with the Gamma variant is significantly higher than that documented for the Alpha variant, for the population younger than 60 years of age and is heterogeneous across age brackets. Our results, both in terms of magnitude and heterogeneity across ages, are aligned with very early evidence on the severity associated with the Gamma variant [14].

Moreover, our sensitivity analysis shows, on the one hand, that vaccination is not a confounding factor for our results. On the other hand, it shows that our assumption on similar vaccine effectiveness against severe illness for the VOCs as for the original virus seems plausible according to Fig 4. This last observation is, indeed, something that needs to be further researched. Similarly, our analysis shows that, while our assumption on constant symptomatic-to-asymptomatic ratios across age brackets is likely affecting the magnitude of our estimates for the relative risk associated with the Gamma variant across age brackets, it does not affect the main insight on its severity. Unfortunately, the data available does not allow us to estimate these ratios for Chile, and what the literature shows is significant differences across regions and time [12]. Our results, though, depend only on the relative ratio across age brackets, and this may display a more stable behavior. This last observation is subject for further research as well.

Our study has several limitations. Overall, the data used by our queueing models was subject to all inaccuracies and biases arising from a data collection process carried out under the most stressful circumstances. In particular, the data available did not have an ideal granularity required to conduct the study in a more straightforward manner, thus we were forced to make assumptions that we would have not made otherwise. For example, while our model assumes that all patients that required an ICU are symptomatic, evidence suggest that this might not always be accurate [15]. Also, we assume similar vaccine effectiveness against severe illness for all variants, and that the symptomatic-to-asymptomatic ratios is homogeneous across age brackets. Also, the distributions of onset to ICU admission and ICU length-of-stay times were calibrated using data collected in the city of Santiago, and thus might not be representative of said times nation-wide. This was also the case, to a lesser extent, of the VOC circulation data, which was sampled across the country without paying particular attention to its representativeness. In addition, there is a subtler underlying assumption about time-homogeneity of the various processes studied, which allow estimation of the various parameters; a more detailed model specification would require much richer data, which at least in the case of Chile, is not publicly available.

Our results are directly relevant for a large portion of the globe where, prior to massive vaccination rollout, the Gamma variant prevailed, such as in Latin America, and for countries where Gamma may have not been dominant but had a large number of cases.

A prime example of the above is Brazil, where Gamma emerged in December 2020 and prevailed until August 2021, and had a slower vaccination rollout, relative to Chile: while 20% of eligible population was vaccinated by early April 2021, in Brazil such figure was not reached until early August 2021 [16]. Argentina, to a lesser extent, also had a slower vaccination rollout while Gamma irrupted. Unfortunately, we could not replicate our findings for the cases of these countries, as the data required to do so is not readily available. In this regard, and despite the limitations listed above, the Chilean government open-access data repository [6], which has been praised internationally [17], turned out essential to carry out the estimation process in this work.

The model presented here may be applied to study the intrinsic severity associated with other VOCs—like the Delta or the Omicron variants—provided that additional and quite demanding data requirements are met. In this regard, our analysis rests on either the assumption of equal vaccine effectiveness against severe illness for the Gamma variant or on the observation of non-vaccinated population. Both assumptions are complex for the cases of Delta an Omicron: as time progressed and new VOCs emerged, in many countries, vaccination rollouts combined vaccines with different technologies (for example, by July 2022, the most common vaccination scheme in Chile consisted in full CoronaVac primary scheme, followed by a BNT612b2 booster, followed by either a BNT612b2 or mRNA-1273 secondary booster [5]). Thus, in the absence of unvaccinated population, the implementation of our model would not only require the estimation of circulation of VOC curves, but also as new VOCs emerge at different stages of the vaccination rollout, new assumptions or data on vaccine effectiveness against severe illness, depending on the VOC and the primary—booster schemed used.

Finally, the model presented here can also be applied to estimate the change in relative risk associated with other endpoints. For example, a modification of our model can be used to study the hypothesis that for patients 40 to 60 years of age, conditional on ICU admission, there is a significant but rather small change in fatality due to the Gamma variant. We posed this hypothesis after observing in the data from the ICU ward at the Hospital Clínico of the Pontificia Universidad Católica, that the ICU fatality due to Gamma (0.21; 7/33) was quite close to that associated with the background strain (0.24; 8/33). There was only one death for a person below 39 in the ICU ward. Considering this, we calibrated a model that explains the overall time-series of fatalities due to COVID-19 using as input the series of mean ICU output by VOC as predicted by the 2021-model (see S4 Appendix for implementation details). As in previous sections, we avoid making assumptions on vaccine effectiveness by focusing the analysis on patients 60 years of age or younger. This analysis showed, indeed, that fatality for the gamma variant was very close to the one from the background strain–and, actually, slightly larger—a result consistent with what was observed in the ICU ward. For the case of people below 39, the exercise delivered a fatality rate for gamma which is less than half the one of the background strain, but we have no actual data from ICU ward to compare to. Overall, this second analysis, which is of course more exploratory than ICU risk, showed that the Gamma variant was overall more severe and deadlier conditional on infection.

## Supporting information

S1 Appendix Estimation of vaccination status of new cases and vaccine effectiveness. (DOCX)

S2 Appendix Estimation of circulation time-series. (DOCX)

S3 Appendix Calibration of onset to ICU and ICU length-of-stay times. (DOCX)

S4 Appendix A first approximation to the effect of the gamma variant on fatality. (DOCX)

S1 Fig Nation-wide new cases time-series by age bracket. Each series has been scaled so that the time average of each series coincides with that of the series associated with patients younger than 40 years of age.
(DOCX)

S1 Table Variant sequencing data. Summary of data on sequencing exams from admitted patients at the Hospital Clínico of the Pontificia Universidad Católica, January to June 2021.
(DOCX)

S2 Table Sensibility analysis computation of probability of ICU conditional on infection caused by background strain. Each column displays the mean square error between the observed and predicted ICU occupancy when the probability of admission conditional to infection is calibrated using data of infection onset during given months (2020).
(DOCX)

S3 Table Mean square error—Comparative fit of the original vs variant-adjusted queueing models. For each age bracket, the mean square error between the expected and observed ICU occupancy during 2020, and during 2021, ignoring and incorporating VOCs.
(DOCX)
