# Supplementary material for: Infection with SARS-CoV-2 variant Gamma (P.1) in Chile increased ICU admission risk three to five-fold
Source: PLoS One. 2023 Mar 24;18(3):e0283085. doi: 10.1371/journal.pone.0283085 (PMC10038273; doi:10.1371/journal.pone.0283085)
Supplement: S1 Appendix — (DOCX) [file pone.0283085.s001.docx]

**S1 Appendix: Estimation of vaccination status of new cases and vaccine effectiveness**

We estimate the vaccination status of new cases considering the effectiveness of the vaccines against contagion. In particular, using Bayes rule, a new case at time *t* belongs to vaccination status *V_s_* with probability

$$P(V_{s}|contagion)=\frac{P(contagion|V_{s})\cdot P(V_{s})}{\sum_{u\in S} P(contagion|V_{u})\cdot P(V_{u})}$$

where S denotes the set of all possible vaccination status (partial or full, with different vaccines, or unvaccinated). We estimate *P(V_s_)* directly from vaccination rollout data [5], for each time *t*. Also, we have that

$$P(contagion|V_{s})=P(contagion|V_{0})\cdot E{f_{s}}^{contagion}$$

where state *V_0_* is “unvaccinated” and the *Ef_s_^contagion^* efficiency factors are taken from government data [4]. Using the above, we have that

$$P(V_{s}|contagion)=\frac{E{f_{s}}^{contagion}\cdot P(V_{s})}{\sum_{u\in S} E{f_{u}}^{contagion}\cdot P(V_{u})}$$

Thus, if *n* new infections occur in time *t* we estimate that *n P(V_s_|contagion)* of them correspond to individuals in vaccination state *V_s_*.

As time progressed, people got vaccinated with different vaccines, thus our model requires the probability of ICU admission conditional on infection for fully and partially vaccinated individuals (that for unvaccinated individuals is estimated directly from pre-vaccination rollout data). For this, we use data on vaccine effectiveness [4]. In particular, we have that for each vaccination status *V_s_* the probabilities of ICU admission and contagion conditional on vaccination status *V_s_* and no vaccination (status *V_0_*) are such that

$$P(ICU|V_{s})=P(ICU|V_{0})\cdot E{f_{s}}^{ICU}, P(contagion|V_{S})=P(contagion|V_{0})\cdot E{f_{s}}^{contagion}$$

where the *Ef_s_^ICU^* and *Ef_s_^contagion^* efficiency factors are taken from government data [4]. Assuming that no uninfected individuals are admitted to the ICU, we have that for all vaccination state *V_s_*

$$P(ICU|V_{S})=P(ICU|V_{s} \wedge contagion)\cdot P(contagion|V_{s})$$

Combining the above, we obtain that

$$P\left( ICU | V_{s}\wedge contagion \right)=\frac{P\left( ICU \right|V_{o}\wedge contagion)\cdot Ef_{s}^{ICU}}{Ef_{s}^{contagion}}$$

With the above, we are able to transform the probability of ICU admission conditional on contagion among unvaccinated individuals into one for vaccinated ones.
