# Supplementary material for: Infection with SARS-CoV-2 variant Gamma (P.1) in Chile increased ICU admission risk three to five-fold
Source: PLoS One. 2023 Mar 24;18(3):e0283085. doi: 10.1371/journal.pone.0283085 (PMC10038273; doi:10.1371/journal.pone.0283085)
Supplement: S2 Appendix — (DOCX) [file pone.0283085.s002.docx]

**S2 Appendix: Estimation of Circulation time-series**

We estimated age-dependent VOC circulation curves using a data set from admitted patients at Hospital Clínico of the Pontificia Universidad Católica. For each of its 1030 entries, the data set contained the age of the patient, and the date and outcome of the sequencing tests. S1 Table presents summary statistics on the data set. Records were aggregated by age-bracket and month; a linear interpolation was used to assign a circulation share for each date between January to June 2021; point estimates were assigned on the 15th of each month, and confidence intervals were produced using a Normal approximation and standard z-scores.
