# Supplementary material for: Infection with SARS-CoV-2 variant Gamma (P.1) in Chile increased ICU admission risk three to five-fold
Source: PLoS One. 2023 Mar 24;18(3):e0283085. doi: 10.1371/journal.pone.0283085 (PMC10038273; doi:10.1371/journal.pone.0283085)
Supplement: S3 Appendix — (DOCX) [file pone.0283085.s003.docx]

**S3 Appendix: Calibration of onset to ICU and ICU length-of-stay times**

**Maximum-Likelihood estimation**

A discrete random variable X is distributed according to a Negative Binomial with mean $\mu$ and dispersion factor r if for any nonnegative integer k, if it fulfills

$$\Pr\left( X=k \right)= \binom{k+r-1}{k}\left( \frac{r}{\mu+r} \right)^{r}\left( \frac{\mu}{\mu+r} \right)^{k}.$$

In our model we assumed that the number of days from infections to admission ($X_{adm}^{a}$) and the duration in ICU ($X_{ICU}^{a}$) are both distributed according to Negative Binomial distributions. Given a data set of durations for a given age bracket a $\left\{ k_{t}^{a}:t\leq T, a\in A \right\}$, we estimated the mean $\mu^{a}$ and dispersion $r^{a}$ of the distribution of X, denoted by $\hat{\mu^{a}}$ and $\hat{r^{a}}$, by maximizing the log-likelihood of the observed data, i.e.

$$\left( \hat{\mu^{a}}, \hat{r^{a}} \right)_{a\in A}\in\arg\max LL= \sum_{a} {LL}_{a}=\sum_{a} \sum_{t\leq T} \log\Pr\left( X=k_{at} \right) .$$

Given our assumption that the durations are independent by age bracket, the log-likelihood is additively separable by a. The Fisher information matrix associated with X, for given values of $\mu$ and r is, then

$$I\left( \mu,r \right)=\left[ \begin{matrix} \frac{r}{\left( \mu+r \right)\mu} & 0 \\ 0 & -E\left( \Psi\left( 1,X+r \right)-\Psi\left( 1,r \right) \right)-\frac{\mu}{\left( \mu+r \right)r} \end{matrix} \right],$$

where expectation is taken with respect to X, and $\Psi$ denotes the second derivative of the log of the gamma function. For a sample of size N, we construct confidence intervals for the parameters using the (asymptotic) Normal approximation

$P\left( \hat{\mu}-\frac{z_{\alpha/2}}{\sqrt{N I_{\mu,\mu}(\hat{\mu},\hat{r})}}\leq\mu\leq\hat{\mu}+\frac{z_{\alpha/2}}{\sqrt{NI_{\mu, \mu}(\hat{\mu},\hat{r})}} \right)\approx Pr\left( \hat{r}-\frac{z_{\alpha/2}}{\sqrt{N I_{r,r}(\hat{\mu},\hat{r})}}\leq r\leq\hat{r}+\frac{z_{\alpha/2}}{\sqrt{NI_{r,r}(\hat{\mu},\hat{r})}} \right)\approx1-\alpha$.

The maximum likelihood estimators of the parameters of the distributions of $X_{adm}^{a}$ and $X_{ICU}^{a}$ for all age brackets are reported in Table 3.

**Hypothesis Testing**

In this section, we describe how we test the hypothesis that onset-to-ICU interval and ICU length-of-stay time distributions do not change for infections with the Gamma and Alpha variants, after and before January 2021.

We considered two data sets for ICU length-of-stay, associated to two variants, $D^{1}= \left\{ k_{n}^{1}:n\leq N^{1},l_{m}^{1}: m\leq M^{1} \right\}$, and $D^{2}=\left\{ k_{n}^{2}:n\leq N^{2},l_{m}^{2}: m\leq M^{2} \right\}$. Each set was composed by a subset $k_{n}^{i}$ of patients who had left the ICU, and a subset $l_{m}^{i}$ of patients remaining in ICU. This distinction is important because the time lengths for $k_{n}^{i}$ represent the actual duration of the process, but $l_{n}^{i}$ is only a lower bound of an event that we have not yet completely observed. We assume that each entry $k_{n}^{i}$ ($l_{m}^{i}$) in our data set $D^{i}$ corresponds to iid (censored) observations from a $NegativeBinomial(\mu^{i},r^{i})$ distribution. Letting $X^{i}$ denote a random variable following such a distribution, the log-likelihood associated with data set $D^{i}$ is given by

$LL\left( D^{i} | \mu^{i},r^{i} \right)=\sum_{n\leq N^{i}} \log\Pr\left( X^{i}=k_{n}^{i} | \mu^{i},r^{i} \right)+\sum_{m\leq M^{i}} \log\Pr\left( X^{i}\geq l_{n}^{i} | \mu^{i},r^{i} \right)$.

To assess if the intervals of different variants follow the same distribution as the original virus, we use the likelihood-ratio test to evaluate if the parameters are equal or (statistically) different. In other words, we test the constraints $\mu^{1}=\mu^{2}=\mu$ and $r^{1}=r^{2}=r.$ The evaluation of this test required the separate estimation of the restricted and unrestricted models to build the $\lambda_{LR}$ statistic.

$$\lambda_{LR}= -2\left( \mathrm{ma}x_{\mu^{1},\mu^{2},r^{1},r^{2}}LL\left( D^{1},D^{2} | \mu^{1},\mu^{2},r^{1},r^{2} \right)-max_{\mu,r}LL\left( D^{1},D^{2} | \mu,r \right) \right)$$

This statistic is compared against a critical threshold, which we set as at 95% confidence, from a $\chi_{(2)}^{2}$ distribution with two degrees of freedom associated to the two aforementioned constraints. The testing strategy for the case of onset-to-ICU interval is simpler as the data is not censored (thus only the first term in the definition of LL is present).

We used the described procedure to test our null hypotheses that time interval distributions for the relevant VOCs follow the same distribution (the restricted model) as the original strain. Table 2A shows maximum likelihood estimates and likelihoods under the restricted and unrestricted models detailed above. From these estimations we fail to reject the null hypotheses that onset-to-ICU interval and ICU length-of-stay distributions are the same (p-values 0.230 and 0.219, respectively). With this result at hand, we proceed to test our null hypotheses that time interval distributions remain unchanged before and after January 2021. Table 2B shows maximum likelihood estimates and likelihoods under the restricted and unrestricted models detailed above. From these estimations we fail to reject the null hypotheses that ICU length-of-stay distributions are the same with 95% confidence.
