# Supplementary material for: Infection with SARS-CoV-2 variant Gamma (P.1) in Chile increased ICU admission risk three to five-fold
Source: PLoS One. 2023 Mar 24;18(3):e0283085. doi: 10.1371/journal.pone.0283085 (PMC10038273; doi:10.1371/journal.pone.0283085)
Supplement: S4 Appendix — (DOCX) [file pone.0283085.s004.docx]

**S4 Appendix: A First Approximation to the Effect of the Gamma Variant on Fatality**

Our main analysis is devoted to understanding the effect of the Gamma variant on the severity of the COVID-19 and its implications on ICU occupancy. However, if we assume that all registered deaths were hospitalized, we can use the number of patients leaving the system as a proxy for the number of deaths. Although it is well documented that a fraction of fatal patients was never hospitalized (Appleby, 2020), our modeling approach can provide a first order approximation of the effect of the Gamma variant on the fatality of the COVID-19. To estimate how the Gamma variant affected fatality, we estimate the following model

$$Y_{t}=\lambda_{bStrain}*X_{t}^{bstrain}+\lambda_{gamma}*X_{t}^{gamma}+\varepsilon_{t}$$

where $Y_{t}$ is the series of registered deaths, $X_{t}^{m}$ is the number of the patients infected with either the background strain (m = bstrain) or the Gamma VOC (m = gamma) that leave the ICU in day t, and $\varepsilon_{t}$ is a normal disturbance with 0 mean and variance σ^2^. In this equation, the parameters $\lambda_{bstrain}$ and $\lambda_{gamma}$ capture the correlation of deaths with the prevalence of the background strain or the Gamma VOC. Note that we have grouped the effect of all prevalent VOC in the Gamma variant, and thus we are estimating the joint effect of the Alpha and Gamma VOCs, understanding that the former variant was not predominant. As in the case of ICU severity, we set the final date in the estimation window for each age bracket to two weeks after the date by which first dose vaccine rollout reached 30% of the eligible population. Unfortunately, this means that for the 60-69 age bracket this alternative estimation was not feasible, because circulation of variants was not large enough to enable identification (that is, the mean square error was insensitive to changes in the risk factor).

To evaluate if the data support the notion of the Gamma variant having a different fatality rate than the background strain, we conducted a Likelihood ratio test, and found that the unrestricted model is preferred (p-val < 0.001). In addition, for each age bracket we conducted an F-test to evaluate if the linear restriction $\lambda_{bstrain}$*=*$\lambda_{gamma}$ holds*.* We reject the null, providing support that the fatality rates are indeed different. With these results at hand, we calibrated the (unrestricted model0): parameter estimates (and standard deviations) are depicted in Table S5 below.

| Age Bracket | *λ_bStrain_* | *λ_gamma_* | LL 2020 | LL 2021 | Test-ratios  (p-value) |
| --- | --- | --- | --- | --- | --- |
| <=39 | 0.383 (0.011) | 0.139 (0.006) | -238.678 | -108.553 | <0.001 |
| 40-49 | 0.459 (0.014) | 0.497 (0.069) | -124.607 | -100.372 | <0.001 |
| 50-59 | 0.465 (0.010) | 0.526 (0.225) | -128.598 | -123.430 | 0.005 |

**S5Table.** Parameter estimates (standard error) and likelihood functions by age bracket.

We observe that there are increases of 8% and 13% in the parameter associated to fatality for the gamma VOC, relative to that associated with the background strain, for age brackets 40-49 and 50-59, respectively. In both cases, we conclude that this change is statistically significant. Our analysis also points to a significant decrease (of about 64%) in the same parameters for the case of patients 39 years of age or younger. While the change in our estimates is aligned with the evidence from the ICU ward data for patients 40 to 60 years of age, we cannot compare the reduction depicted in Table S5 for patients 39 years of age or younger against observations of the ICU ward data, as the number of deaths in this age bracket is too small (we observed only one death in this age bracket).

**References**

Appleby J. What is happening to non-covid deaths? *BMJ*. 2020;369:m1607. Published 2020 Apr 24. doi:10.1136/bmj.m1607
