# Supplementary material for: Infection with SARS-CoV-2 variant Gamma (P.1) in Chile increased ICU admission risk three to five-fold
Source: PLoS One. 2023 Mar 24;18(3):e0283085. doi: 10.1371/journal.pone.0283085 (PMC10038273; doi:10.1371/journal.pone.0283085)
Supplement: S1 Fig — Each series has been scaled so that the time average of each series coincides with that of the series associated with patients younger than 40 years of age. (DOCX) [file pone.0283085.s005.docx]

**
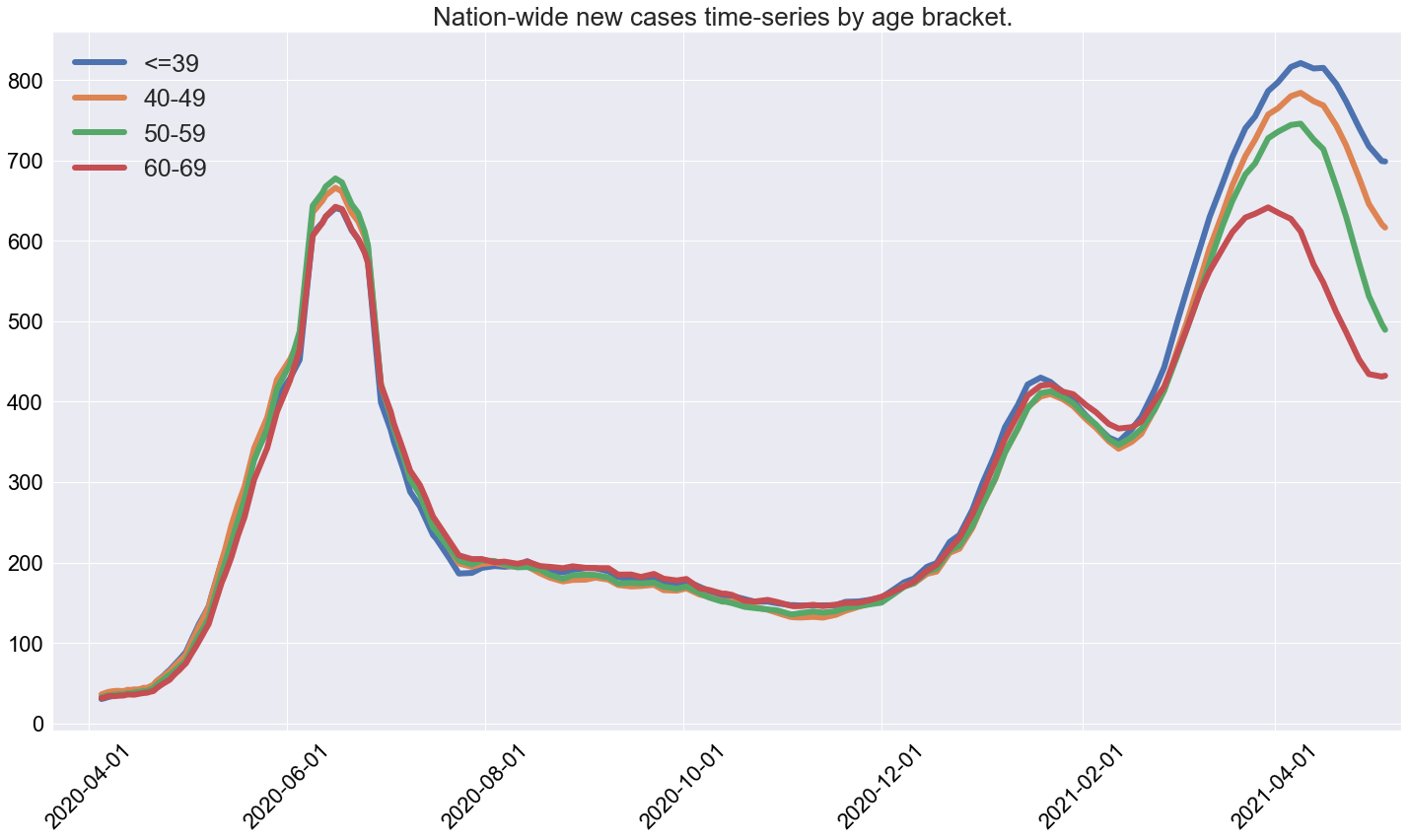
**

**S1 Figure. Nation-wide new cases time-series by age bracket.** Each series has been scaled so that the time average of each series coincides with that of the series associated with patients younger than 40 years of age.
