# Supplementary material for: Infection with SARS-CoV-2 variant Gamma (P.1) in Chile increased ICU admission risk three to five-fold
Source: PLoS One. 2023 Mar 24;18(3):e0283085. doi: 10.1371/journal.pone.0283085 (PMC10038273; doi:10.1371/journal.pone.0283085)
Supplement: S1 Table — Summary of data on sequencing exams from admitted patients at the Hospital Clínico of the Pontificia Universidad Católica, January to June 2021. (DOCX) [file pone.0283085.s006.docx]

| **Age-bracket** | **January** | | | **February** | | | **March** | | | **April** | | | **May** | | |
| --- | --- | --- | --- | --- | --- | --- | --- | --- | --- | --- | --- | --- | --- | --- | --- |
|  | **P1** | **B117** | **others** | **P1** | **B117** | **others** | **P1** | **B117** | **others** | **P1** | **B117** | **others** | **P1** | **B117** | **others** |
|  |  |  |  |  |  |  |  |  |  |  |  |  |  |  |  |
| **<=39** | 0 | 0 | 15 | 3 | 3 | 47 | 33 | 9 | 37 | 79 | 15 | 76 | 133 | 6 | 70 |
| **40-49** | 0 | 0 | 8 | 0 | 1 | 15 | 7 | 3 | 16 | 26 | 5 | 21 | 51 | 0 | 18 |
| **50-59** | 0 | 0 | 1 | 0 | 0 | 14 | 5 | 0 | 9 | 30 | 4 | 24 | 12 | 0 | 11 |
| **60-69** | 0 | 0 | 3 | 0 | 0 | 8 | 6 | 4 | 3 | 11 | 2 | 7 | 18 | 0 | 4 |

**S1 Table.** **Variant sequencing data**. Summary of data on sequencing exams from admitted patients at the Hospital Clínico of the Pontificia Universidad Católica, January to June 2021.
