# Supplementary material for: Infection with SARS-CoV-2 variant Gamma (P.1) in Chile increased ICU admission risk three to five-fold
Source: PLoS One. 2023 Mar 24;18(3):e0283085. doi: 10.1371/journal.pone.0283085 (PMC10038273; doi:10.1371/journal.pone.0283085)
Supplement: S2 Table — Each column displays the mean square error between the observed and predicted ICU occupancy when the probability of admission conditional to infection is calibrated using data of infection onset during given months (2020). (DOCX) [file pone.0283085.s007.docx]

| **Age bracket** | **May** | **June** | **July** | **August** | **September** | **October** | **November** | **December** |
| --- | --- | --- | --- | --- | --- | --- | --- | --- |
|  |  |  |  |  |  |  |  |  |
| **<=39** | 1212.86 | 1210.88 | 1573.99 | 1271.69 | 1203.43 | 1193.9 | 1204.45 | 1254.07 |
| **40-49** | 2017.77 | 2077.12 | 2894.38 | 2154.53 | 1987.55 | 1993.02 | 2038.42 | 2452.11 |
| **50-59** | 8319.44 | 8557.41 | 14764.47 | 10914.99 | 8775.82 | 8405.30 | 8507.70 | 12681.39 |
| **60-69** | 11032.79 | 11277.67 | 24479.38 | 18020.92 | 13380.20 | 12046.73 | 11025.33 | 14158.47 |

**S2 Table**. **Sensibility analysis computation of Probability of ICU conditional on infection caused by background strain.** Each column displays the mean square error between the observed and predicted ICU occupancy when the probability of admission conditional to infection is calibrated using data of infection onset during given months (2020).
