# Supplementary material for: Infection with SARS-CoV-2 variant Gamma (P.1) in Chile increased ICU admission risk three to five-fold
Source: PLoS One. 2023 Mar 24;18(3):e0283085. doi: 10.1371/journal.pone.0283085 (PMC10038273; doi:10.1371/journal.pone.0283085)
Supplement: S3 Table — For each age bracket, the mean square error between the expected and observed ICU occupancy during 2020, and during 2021, ignoring and incorporating VOCs. (DOCX) [file pone.0283085.s008.docx]

| Age-bracket | 2020 | 2021 | |  |
| --- | --- | --- | --- | --- |
|  |  | No *VOC* | *VOC* |  |
| <=39 | 302.96 | 22228.17 | 509.90 |  |
|  |  |  |  |  |
| 40-49 | 433.05 | 41998.27 | 263.31 |  |
|  |  |  |  |  |
| 50-59 | 826.57 | 59198.14 | 1642.75 |  |
|  |  |  |  |  |
| 60-69 | 2551.59 | 12302.86 | 2219.31 |  |
|  |  |  |  |  |

**S3 Table. Mean square error - comparative fit of the original vs variant-adjusted queueing models.**  For each age bracket, the mean square error between the expected and observed ICU occupancy during 2020, and during 2021, ignoring and incorporating VOCs.
